# A case study of cost‐benefit analysis in occupational radiological protection within the healthcare system of Sweden

**DOI:** 10.1002/acm2.13421

**Published:** 2021-09-10

**Authors:** Andreas Engström, Mats Isaksson, Reza Javid, Charlotta Lundh, Magnus Båth

**Affiliations:** ^1^ Department of Radiology Skaraborg Hospital Skövde Sweden; ^2^ Department of Radiation Physics Institute of Clinical Sciences Sahlgrenska Academy at University of Gothenburg Gothenburg Sweden; ^3^ Department of Research and Development Skaraborg Hospital Skövde Sweden; ^4^ Department of Medical Physics and Biomedical Engineering Sahlgrenska University Hospital Gothenburg Sweden

**Keywords:** ALARA principle, α‐value, cost‐benefit analysis, healthcare, monetary cost assigned to a unit of collective dose, occupational radiological protection

## Abstract

The aim of the present study was to demonstrate cases of cost‐benefit analysis within healthcare, of how economic factors can be considered in occupational radiological protection, in agreement with the as low as reasonably achievable principle and present Swedish legislations.

In the first part of the present study, a comparison of examples within health economics used by authorities and institutes in Sweden was made. The comparison focused on value of a statistical life, quality‐adjusted life year, and monetary cost assigned to a unit of collective dose for radiation protection purposes (α‐value). By this comparison, an α‐value was determined as an interval between $45 and $450 per man‐mSv, for the Swedish society in 2021. The α‐value interval can be interpreted as following:

1. Less than $45 per man‐mSv is a good investment.
2. From $45 to $450 per man‐mSv, other factors than costs and collective dose are important to consider.
3. More than $450 per man‐mSv is too expensive.

In the second part of the present study, seven cases of cost‐benefit analyses in occupational radiological protection were provided. The present study focused specifically on cases where the relevant factors were costs and collective dose. The present case study shows a large variation in costs per collective dose from different types of occupational radiological protection, used at Skaraborg Hospital in Sweden.

## 1 INTRODUCTION

Good knowledge about the risks of ionizing radiation is important in order to make good judgments in occupational radiological protection. To evaluate the economic costs associated with occupational radiological protection, the risks of ionizing radiation must also be compared with other risks in society. For instance, the average loss of life expectancy is lower for the Japanese atomic bomb survivors who were in the most exposed group (mean 2.25 Gy) than for severe obesity (body mass index over 40) or smoking (18 cigarettes per day).

The as low as reasonably achievable (ALARA) principle can be traced back to the International Commission on Radiological Protection (ICRP) Publication 9,^2^ where it was expressed as: “As any exposure may involve some degree of risk, the commission recommends that any unnecessary exposure be avoided and that all doses be kept as low as is readily achievable, economic and social considerations being taken into account.” In ICRP Publication 22,^3^ the meaning of this earlier statement was reviewed, and the word readily was replaced by reasonably. The ALARA principle is included in both The Principle of Optimization of Protection in ICRP Publication 103^4^ and in the European Union council directive on basic safety standards, Article 5.^5^ The directive forces the member states of the European Union to include the ALARA principle in their national legislations. According to Huda et al.^6^ the ALARA principle has a laudable intent, but in practice it is often problematic. For example, it is obvious that lead aprons used in nuclear medicine bring dose reduction to staff, but it is not obvious in which situations lead aprons are reasonable to use.^6^

How assessments in optimization should be carried out in agreement with the ALARA principle has been described in several reports, and one of several options in the optimization process is to use cost‐benefit analysis, where the detriment is presented in monetary terms and compared with the costs of radiation protection.^3^, ^7^, ^8^, ^9^, ^10^, ^11^, ^12^, ^13^ Optimization is to balance the costs with the benefits from radiation protection, therefore the best option is not always the one with the lowest dose.^10^, ^13^ Radiation protection always comes with a cost for society in terms of work, materials, and sometimes risks.^8^ To evaluate the cost of the detriment of ionizing radiation, a monetary term has been introduced, which describes the monetary cost assigned to a unit of collective dose for radiation protection purposes (the α‐value).^3^, ^8^, ^10^ In many situations, a good approximation to the cost of the detriment (Y) can be expressed as Y  =  αS, where S is the collective dose. This formula can be used in a cost‐benefit analysis to find the optimum level of protection.^3^, ^8^, ^10^ According to ICRP,^9^ cost‐benefit analysis is best used in situations where the relevant factors involved are possible to quantify in monetary terms, for example costs and collective dose. If other factors are relevant (that are not easy to quantify), a more qualitative solution is preferable.^9^, ^11^, ^14^

In the literature, several examples^3^, ^8^, ^10^, ^14^, ^15^, ^16^, ^17^, ^18^, ^19^ of α‐values can be found. For the purpose of data comparison in the present study, α‐values have been adjusted for inflation,^20^ rounded, and (where applicable) converted to USD per man‐mSv. In 1973, ICRP^3^ presented that α‐values from previous literature were in the range from $6 to $150 per man‐mSv. Ten years later (in 1983), ICRP^8^ presented that α‐values from previous literature now were in the range from $2.7 to $270 per man‐mSv. However, ICRP^8^ also indicated that a narrower range from $27 to $54 per man‐mSv would be more realistic. In 1995, the U.S Nuclear Regulatory Commission^15^ recommended an α‐value of $350 per man‐mSv. This conclusion was partly based on a study by Baum,^16^ which describes a wide range of estimates of values of a statistical life. In 2002, the International Atomic Energy Agency (IAEA)^14^ presented a summary of recommended α‐values from different national authorities, in the range from $110 to $4 430 per man‐mSv. In 2014, the US Department of Energy^10^ recommended an α‐value as an interval for the public from $110 to $670 per man‐mSv. If the α‐value is set to $670 per man‐mSv, and the risk of fatal cancer is assumed to be 5 x 10^‐5^ per man‐mSv per man‐mSv, the recommended value would thus equate $13.4 million per hypothetical radiation‐induced cancer death averted.^10^ According to a study by Andresz et al. in 2020,^18^ 80% of the nuclear reactors worldwide were using the concept of an α‐value in their optimization processes. In that survey, α‐values varied from $540 to $6 100 per man‐mSv. This is a range of values considerably higher than the recommendation from the US Department of Energy,^10^ but in closer agreement with the α‐values from different national authorities published by IAEA.^14^ The above examples indicate that α‐values have increased over the years (the α‐values described are adjusted for inflation). Also, from the examples, α‐values are sometimes expressed as an interval to manifest the large uncertainty around its valuation.

Official monetary valuation of a road accident fatality in 2002 was $3.0 million for the US, $1.9 million for Sweden, and $0.05 million for Portugal.^21^ One of the reasons for this variation is the difference in gross domestic product per capita between countries.^21^ Hence, α‐values defined by authorities in other countries cannot directly be implemented in a given society.^8^, ^19^

In Sweden in 1992, the Swedish Radiation Safety Authority recommended an α‐value of $150 per man‐mSv,^19^ adjusted for inflation.^22^ In the past years, the nuclear utilities in Sweden have all reported α‐values close to $1 300 per man‐mSv in their optimization processes,^18^ which is a factor of 10 higher than the recommendation from the Swedish Radiation Safety Authority in 1992.

In this context, legislations of individual dose limits should always be respected, regardless the result of a cost‐benefit analysis.^7^, ^19^ The purpose of a dose limit is to restrict the occurrence of stochastic effects of ionizing radiation to an acceptable level. This level of acceptance was in 1977 referred to as an average annual risk of mortality less than 10^–4^, which could be seen in other occupations with high standards of safety, where the workers were not exposed to ionizing radiation.^7^

Neither the European Union council directive on basic safety standards^5^ nor Swedish legislations^23^, ^24^, ^25^, ^26^ present any constraints on using specific radiation protection tools. However, in other national legislations, this type of constraints may exists, and the ALARA principle would then be overruled in those specific cases.

The aim of the present study was dual. First, the aim was to compare examples within health economics used by authorities and institutes in Sweden, and by these examples calculate corresponding α‐values for radiation protection purposes. In this way, a recommendation of an α‐value could be determined for the Swedish society in 2021. Since most of the examples of cost‐benefit analysis in radiation protection are related to the nuclear industry,^10^, ^12^, ^18^ the second part of the aim of the present study was to describe cases of cost‐benefit analysis in occupational radiological protection within the healthcare system of Sweden.

The present study demonstrates cases of cost‐benefit analysis within healthcare, of how economic factors can be considered in occupational radiological protection, in agreement with the ALARA principle and present Swedish legislations.

## 2 METHODS

Ethical review of the present study was waived by the Swedish Ethical Review Authority.

In cost‐benefit analysis within health economics, it is important to consider the social discount rate, which is used to calculate the present value of a consequence in the future. If a high social discount rate is used, small considerations are taken for consequences in the future.^27^ One reason to use social discount rate in health economics is that societies often grow healthier over time.^28^ Another reason is that people are impatient and prefer advantages now rather than later.^28^ In health economics in Sweden, a social discount rate of 3% is usually applied.^27^

Quality‐adjusted life year (QALY) is a concept that is frequently used in healthcare. One QALY is defined as one person living 1 year with perfect health.^27^ When new drugs are evaluated, they can be compared with present alternatives by calculating the differences in price per QALY.^29^

The value of a statistical life (VSL) is the willingness of the society to pay to prevent the death of one statistical person.^27^ The calculations of VSL are often based on surveys, where people are asked how much they are willing to pay to avoid a small risk of death. By answering this question people also give an answer about the value of a statistical person, which is called the stated preference approach of willingness to pay. The estimates of VSL applie to non‐identified persons that are exposed to a risk close to zero.^27^ VSL is therefore applicable to the cancer‐risk of small doses of ionizing radiation.

Studies have shown that estimates of VSLs are depended on the context of risk.^16^, ^30^ In the present study, an attempt has been made to reduce these differences in the Swedish society. Therefore, a comparison of three Swedish authorities and institutes were made, focusing on QALYs, VSLs, and α‐values in 2021 price levels:^22^

- In 2012, the Swedish Civil Contingencies Agency published a report on the economical assessment of present and future health risk in Sweden.^27^ In this report, the value of VSL ranges from $1.1 to $10.1 million, and the value of a QALY ranges from $0.05 to $0.22 million. The statement of VSL in Sweden is based on an empirical literature review by Hultkrantz and Svensson.^30^ Most of the references in this literature review are based on the stated preference approach within road accidents.
- In 2016, the Swedish Institute for Health Economics published a report on the willingness to pay for decreasing the risk of morbidity and mortality within road accidents.^31^ This report shows a VSL of $4.1 million and QALYs in a range from $0.16 to $0.57 million. Based on this report and other studies, the Swedish Transport Administration has decided that the VSL in the Swedish transport sector should be $4.4 million.^32^
- In 2015, Svensson et al.^29^ published a study, which showed that the limit for The Swedish Dental and Pharmaceutical Benefits Agency of accepting a drug in the national pharmaceutical benefits scheme was between $0.11 and $0.18 million per QALY. The National Board of Health and Welfare in Sweden has described the costs for a QALY below $0.01 million as low and costs over $0.1 million as very high.^33^

For the reported QALYs from The Swedish Dental and Pharmaceutical Benefits Agency, the work by Persson and Hjelmgren^34^ was used to derive VSLs out of those reported QALYs. Persson and Hjelmgren^34^ have described a relation between VSLs from traffic‐situations in Sweden and QALYs within the healthcare system, by considering discount rate, quality of life, and taxes.

Mubayi et al.^17^ have calculated the medical costs and loss of VSLs from different types of radiation‐induced cancers, looking at both latency periods and incidence rates (mortality and morbidity) at different discount rates. According to their work (with a 3% discount rate), a VSL of $3 million corresponds to an α‐value of $122 per man‐mSv. By prorating this value, other α‐values can be derived from VSLs.^17^ This approach was used to derive α‐values of all the reported VSLs from the three Swedish authorities and institutes described above.

In this way, an interval of α‐values could be determined for the Swedish society in 2021. A recommendation of investments in radiation protection could then be interpreted from this interval. The recommendation was divided into three groups by applying the “band scheme” from Croft and Lochard^35^:

1. A good investment.
2. Other factors than costs and collective dose are important to consider.
3. Too expensive.

A total of seven cases of cost‐benefit analyses in occupational radiological protection from Skaraborg Hospital were provided; five cases within radiology and two cases within nuclear medicine. The present study focused specifically on cases where the relevant factors were costs and collective dose. Realistic assumptions, rather than worst‐case‐scenarios, were used in the present study, in accordance with the US Department of Energy and IAEA.^10^, ^14^ The seven cases were evaluated by using the recommendation determined in the present study, concerning the α‐value for the Swedish society in 2021.

## 3 RESULTS

Table 1 shows a comparison of three key references focusing on QALYs, VSLs and α‐values in 2021 price levels. Because of the uncertainties in the estimations of VSL, the Swedish Civil Contingencies Agency presents its recommendations as an interval, with a factor of 10 between low and high.^27^ This interval agrees with the other two references in Table 1.^29^, ^31^ As mentioned in the introduction section, the Swedish Radiation Safety Authority^19^ assigned costs to collective dose in 1992, these costs correspond to $150 per man‐mSv in 2021 price levels, also in agreement with the interval from the Swedish Civil Contingencies Agency.^27^ Worth noting, the reported α‐values used by the nuclear utilities in Sweden^18^ (close to $1 300 per man‐mSv) are higher than the interval from the Swedish Civil Contingencies Agency.^27^ Even so, in the present study, the conclusion of the figures in Table 1 was that the interval of VSLs from the Swedish Civil Contingencies Agency^27^ is useful as a recommendation of the α‐value for the Swedish society in 2021. As mentioned in the methods section, the recommendation of investments in radiation protection was interpreted from the α‐value interval. The recommendation was divided into three groups and is presented in Table 2.

**TABLE 1:** Comparison of three key references focusing on QALYs, VSLs, and α‐values in 2021 price levels

	QALY	VSL	α‐value
Swedish authorities and institutes	(millions of USD)	(millions of USD)	(USD per man‐mSv)
The Swedish Civil Contingencies Agency	0.05–0.22	1.1–10.1	45–450^b^
The Swedish Institute for Health Economics	0.16–0.57	4.1	170^b^
The Swedish Dental and Pharmaceutical Benefits Agency	0.11–0.18	2.7–4.5^a^	110–180^b^

Abbreviations: QALY, quality‐adjusted life year; VSL, value of a statistical life.

^a^ VSL was derived from QALY by using the work of Persson and Hjelmgren^34^ ^.^

^b^ α‐value was derived from VSL by using the work of Mubayi et al.^17^ ^.^

**TABLE 2:** Recommendation of investments in radiation protection interpreted from the α‐value interval for the Swedish society

α‐value	
(USD per man‐mSv)	Recommendation
<45	A good investment
45–450	Other factors than costs and collective dose are important to consider.
>450	Too expensive

In the following section, a total of seven cases of cost‐benefit analyses in occupational radiological protection from Skaraborg Hospital are provided; five cases within radiology and two cases within nuclear medicine.

### 3.1 Real‐time staff dosimeters

Several studies have shown promising dose reduction for the staff when they started working with real‐time dosimeters. In cerebral angiography, a dose reduction of up to 70% for a physician and 50% for an assisting nurse could be seen.^36^ In cardiology, a dose reduction up to 59% could be seen for a cardiologist, and for the assisting nurses as a group, the dose reduction was around 40%.^37^ Pain physicians received a dose reduction of 46 % with a combination of real‐time dosimeters and active coaching.^38^

In 2012, two systems with a total of 20 real‐time staff dosimeters were installed at the department of Interventional Radiology at Skaraborg Hospital. The total cost of these systems was $56,000. The systems were expected to be used for 10 years, and the collective dose saved during these years was estimated to be around 70 man‐mSv. This incurs a cost of $800 per man‐mSv, which according to the recommendations in Table 2 is too expensive.

### 3.2 X‐ray overhead shields

In 2014, a supplier presented a new model of an overhead shield, with an overlapping panel curtain and radiation protective drapes for femoral and radial access. The effective dose of the examiner varies greatly with the height above the floor, at 140 cm the dose reduction with the new model (with an overlapping panel curtain, but without protective drapes) gives a dose reduction of 68.4% ± 7.4% compared with the suppliers old model.^39^ The collective dose of the cardiologists (and their assistants) working with the old model of overhead shield at Skaraborg Hospital was measured at breast height (under the lead aprons) to be 0.3 man‐mSv during 2019 (in one examination room). The new model of overhead shield was assumed to be used for 10 years.

The cost for the new shield (without sterile disposable covers for femoral and radial access) was $4,000. This gives a cost of $1,900 per man‐mSv, which according to the recommendations in Table 2 is too expensive.

If radiation protective drapes for femoral and radial access (with sterile disposable covers) were purchased, the dose reduction would be higher but the costs per collective dose would also be higher.

According to ICRP,^40^ the threshold dose for radiation‐induced cataract is 0.5 Gy. The equivalent dose to the eyes of cardiologists at Skaraborg Hospital was estimated to be far below the dose limit (20 mSv per year) recommended by the ICRP^40^ working with their old model of overhead shield. Therefore, the dose reduction at eye‐level with the new overhead shield compared with their old model was not considered a relevant factor.

### 3.3 Lead aprons in cardiology and interventional radiology

The cost for a lead apron on the Swedish market in 2019 was about $590. From experience at Skaraborg Hospital, a lead apron that is regularly worn can be used for 5 years before it must be disposed.

If the costs of a lead apron should not be too expensive, according to the recommendations in Table 2 (more than $450 per man‐mSv), the use of lead apron needs to avert a collective dose of more than 0.26 man‐mSv per year. In the same way, if the use of lead apron is averting a collective dose of more than 2.6 man‐mSv per year, it is a good investment. In the interval between these figures, other factors than costs and collective dose are important to consider.

The dose reduction of using a lead apron compared to not using one can be approximated to be 90 %, in cardiology and interventional radiology.^41^ At Skaraborg Hospital (in cardiology and interventional radiology), 10 of 29 lead aprons had an estimated collective dose reduction of less than 0.26 man‐mSv per year. These aprons are therefore too expensive according to the recommendations in Table 2. Of the 29 aprons, 12 were in the range where other factors than costs and collective dose are important to consider, and seven were a good investment.

The lead aprons in cardiology and interventional radiology at Skaraborg Hospital are used as personal aprons. If an apron instead were being used by several members of the staff, the costs per collective dose would be reduced, and the calculations would lean into a better investment, an observation also reported by Russel and Hufton.^42^

### 3.4 Thyroid collars in cardiology and interventional radiology

The costs for a thyroid collar on the Swedish market in 2019 were about $100, and the thyroid collar was estimated to be used for 5 years before disposal.

For the thyroid collar not to be too expensive, according to the recommendations in Table 2 (more than $450 per man‐mSv), the use of thyroid collar needs to avert a collective dose of more than 0.044 man‐mSv per year. In the same way, if the use of thyroid collar is averting a collective dose of more than 0.44 man‐mSv per year, it is a good investment. In the interval between these figures, other factors than costs and collective dose are important to consider.

The tissue weighting factor for the thyroid is estimated to be 0.04.^4^ The protection factor for a thyroid collar of 0.35 mm Pb has been estimated to be around 7 in cardiology and interventional radiology.^43^, ^44^ By using the tissue weighting factor for the thyroid and the protection factor of a thyroid collar, 15 of 29 thyroid collars (used in cardiology and interventional radiology at Skaraborg Hospital) had an estimated collective dose reduction of less than 0.044 man‐mSv per year. These thyroid collars are therefore too expensive according to the recommendations in Table 2. Of the 29 thyroid collars, 11 were in the range where other factors than costs and collective dose are important to consider, and three were a good investment.

The thyroid collars in cardiology and interventional radiology at Skaraborg Hospital are used as personal collars. In the same way as for lead aprons, the costs per collective dose would be reduced if the thyroid collars were shared among several staff members.

### 3.5 Radiation protective gloves

Radiation protective gloves are used to protect the hands from scattered secondary radiation during fluoroscopic procedures. The gloves are not intended to be used in the primary X‐ray beam. According to one manufacturer, the lead equivalence is 0.032 mm in their protective gloves (thin surgical gloves of latex), and the attenuation is 53 % (80 kVp), figures that have been confirmed by measurements at Skaraborg Hospital. In 2015, equivalent doses to the hands of physicians (Hp 0.07) were measured at Skaraborg Hospital. The mean annual estimated equivalent dose to a hand was around 26 mSv, without radiation protective gloves. The limit of the equivalent dose to the skin is 500 mSv in 1 year,^5^ none of the physicians were close to the dose limit, and therefore there are no concerns of deterministic effects. The effective dose from the scattered radiation to the skin of the hands was calculated by taking the ratio of the skin area of the two hands over the whole skin area (0.018) and multiplying with the tissue weighting factor 0.01.^4^, ^45^, ^46^ The effective dose that emerges from exposure of the hands was estimated to be around 5 μSv in a year. If it is assumed that the physician's hands are not in the primary beam and that radiation protective gloves could be used at all times, the effective dose that emerges from exposure of the hands could be halved.

The gloves are used in a sterile environment and are therefore discarded after each procedure. On the Swedish market (in 2015) radiation protective gloves could cost $35 per pair. The physicians using these protective gloves at Skaraborg Hospital performed around 250 procedures per year. This brings the costs to around $3.7 million per man‐mSv, which is too expensive according to the recommendations in Table 2.

### 3.6 Lead aprons in the department of nuclear medicine

In 2019, two new lead aprons (0.5 mm Pb) were purchased to be shared among seven employees at the department of nuclear medicine; the cost was $1,300.

The lead aprons were assumed to be used for 5 years before they would be replaced. The effective dose for the seven employees is regularly estimated, and their collective dose was 4 man‐mSv per year. The use of a lead apron in a department of nuclear medicine (compared to not using a one) is estimated to reduce the effective dose by a half.^47^, ^48^

The cost per collective dose was $130 per man‐mSv, which is in the interval where other factors than costs and collective dose are important to consider, according to the recommendations in Table 2.

### 3.7 Lead shielding of a drywall in the department of nuclear medicine

In 2016, a new SPECT‐CT was installed at Skaraborg Hospital. The drywall between the examination room and the controlled area of the radiology reading room had at the moment of installation no lead shielding. The examination room was not expected to be used by the department of nuclear medicine for longer than 10 years. The effective dose for the radiologists, when working in the radiology reading room, was estimated. The collective dose was assumed to be no higher than 22 man‐mSv in the coming 10 years.

The cost for installation of 1‐mm lead shield on the drywall was $3,500. The 1‐mm lead was calculated to attenuate 92% of the scattered radiation from technetium‐99 m.^49^ The amount of scattered radiation from other nuclides and from X‐rays was calculated to be very small.

The cost per collective dose was $170 per man‐mSv, which is in the interval where other factors than costs and collective dose are important to consider according to the recommendations in Table 2.

In Table 3, the results of the present case study of cost‐benefit analysis in occupational radiological protection within the healthcare system of Sweden are presented. The specific assumptions that are made in each case are crucial for the result. Therefore, other assumptions can lead to a different outcome of a case. The costs per collective dose were not recalculated into 2021 price levels, since it would have a negligible effect on the outcome of the case. The results of the present case study show a large variation of costs per collective dose from different types of occupational radiological protection.

**TABLE 3:** Cost‐benefit analysis in occupational radiological protection within the healthcare system of Sweden

Case	Costs per collective dose (USD per man‐mSv)	Outcome of cost‐benefit analysis
3.1 Real‐time staff dosimeters	800	Too expensive
3.2 X‐ray overhead shields	1,900	Too expensive
		10 of 29 aprons were too expensive.
3.3 Lead aprons in cardiology and interventional radiology	–	2. 12 of 29 aprons, other factors than costs and collective dose are important to consider.
		3. 7 of 29 aprons were a good investment.
		1. 15 of 29 collars were too expensive.
3.4 Thyroid collars in cardiology and interventional radiology	–	2. 11 of 29 collars, other factors than costs and collective dose are important to consider.
		3. 3 of 29 collars were a good investment.
3.5 Radiation protection gloves	3,700,000	Too expensive
3.6 Lead aprons in the department of nuclear medicine	130	Other factors than costs and collective dose are important to consider.
3.7 Lead shielding of a drywall in the department of nuclear medicine	170	Other factors than costs and collective dose are important to consider.

## 4 DISCUSSION

The present study demonstrates cases of cost‐benefit analysis within healthcare, of how economic factors can be considered in occupational radiological protection, in agreement with the ALARA principle and present Swedish legislations. An α‐value for the Swedish society in 2021 was presented as an interval between $45 and $450 per man mSv. The present case study shows a large variation in costs per collective dose from different types of occupational radiological protection, used at Skaraborg Hospital in Sweden.

In cost‐benefit analysis in radiation protection, uncertainties in estimations of collective dose can sometimes be of significance. The interpretation from the α‐value interval in the present study was divided into three groups: a good investment, other factors than costs and collective dose are important to consider, and too expensive. A factor of 10 was used for the interval of costs per collective dose for the mid alternative. In this way, despite uncertainties in estimations of collective dose, the outcome of the cases in the present study is not likely to go from a good investment into a too expensive one and vice versa. The outcome of the cases, therefore, is not very sensitive to uncertainties of collective dose.

A position in radiation protection is that costs assigned to a unit of collective dose for radiation protection purposes should increase with the level of individual exposure instead of being constant.^8^, ^14^, ^50^ To describe this situation, besides the α‐value, an extra term in cost‐benefit analysis was introduced as the β‐value.^8^, ^14^ However, in this position, under a certain level of individual exposure it is appropriate to exclude the β‐value, and only use the α‐value. In occupational exposure, this level is suggested to be 1 mSv per year,^12^, ^14^, ^50^ the same as the dose limit for the public,^5^ a criterion which is met by most of the cases in the present study.

If the individual dose distribution within a group is inhomogeneous, the concept of collective dose could be misleading when used in a cost‐benefit analysis. To overcome such a situation, collective doses can be estimated for sub‐groups with similar characteristics.^13^ In the present study, all the estimated individual effective doses were below 1 mSv per year and were therefore considered to have rather homogeneous characteristics.

The α‐value presented in the present study is useful in cases where the benefit of one option of occupational radiological protection is valuated in relation to its costs, which is often the case within healthcare. However, different options of radiation protection can be compared with each other by simply comparing their ratios of costs and dose reduction in a so‐called cost‐effectiveness analysis.^9^, ^10^, ^13^ It has been shown that when evaluating different options in comparison to each other, the process is not very sensitive to the α‐value.^10^

The stated preference approach of VSL has many drawbacks.^17^, ^30^ One of the concerns is the scale insensitivity, which shows that respondents have difficulties with the magnitude of the risk.^51^ Respondents’ willingness to pay is expected to be proportionally to the magnitude of the risk they want to avoid, but this is often not the case.^51^ Another concern is that respondents in surveys tend to overestimate their willingness to pay compared to situations in real life.^52^ Furthermore, the concept of VSL might be depended on age.^53^, ^54^ The US Environmental Protection Agency proposed an age‐dependent VSL with a senior discount in 2002. This proposal generated a political firestorm and was therefore abandoned.^53^ In health economics, an average VSL of the entire population is often used,^53^ which was also the choice in the present study. However, many studies have shown that VSL does vary with age. One model is the inverted U‐shape where VSL peaks around age 40.^53^ Another model is that avoiding a fatality of a child is worth more than avoiding a fatality of an adult.^54^ As mentioned in the result, the Swedish Transport Administration recommends a VSL of $4.4 million.^32^ This VSL is partly based on the mean loss of QALYs in a road accident,^32^ which indicates a VSL dependent on age.

In the stated preference approach of VSL, some studies have shown that VSLs are almost the same in different contexts of risk,^54^ whereas other studies have shown the opposite results.^16^, ^30^ For example, in situations where the magnitude of the risk is constant, the respondents can value a higher VSL for airline safety compared to highway safety.^16^, ^30^ Another comparison reported by Baum^16^ was a VSL of $0.26 million for highway safety compared to a VSL of $15 million for radiation‐related activities. Baum^16^ also reported that different authorities in the US use different values of VSLs. In the recommendation of the α‐value in the present study, comparisons of VSL‐estimations from different Swedish authorities and institutes were made. In this way, an attempt was made to reduce the differences between contexts of risk. One universal VSL could help society to allocate resources equally between different contexts of risk. In reality, politicians often allocate resources based on their own beliefs and the media impact.^19^

In th estimations of VSL, there is a discrepancy between the public and workers.^17^ A comparison have shown that the mean‐estimated VSL for the public ($11 million) is a factor of 1.2 higher than the mean‐estimated VSL for workers ($9 million).^17^ ICRP^4^ also determines the risk factor for stochastic effects, after exposure to radiation at low dose rate, to be 1.3 higher for the whole population compared to adults (the working age population), because of the age differences in these groups. These two differences between the public and workers in combine (1.56) can still be valued as small compared to the overall uncertainties in estimations of VSLs. The US Department of Energy promotes an interval (with a factor of 6) of the α‐value in their recommendation, as a consequence of these uncertainties.^10^ As mentioned in the methods section, an empirical literature review by Hultkrantz and Svensson^30^ presented that most of the estimations of VSLs in the Swedish society could be included in an interval with a factor of 10. This interval can be seen as the minimum and maximum of a reliable estimation of VSL. Therefore, the interval was considered to be large enough to embrace the rather small discrepancy (1.56) between the public and workers. In this context, an interval with a factor of 10 of the α‐value was chosen to be the best option in the present study, in agreement with the interval of VSLs recommended by The Swedish Civil Contingencies Agency.^27^

In the end, despite all the drawbacks and uncertainties with the stated preference approach of VSL described above, many economists see these methods as the best available to deal with small risks within society.^17^ Having good estimations of VSLs has become more important as cost‐benefit analyses are increasingly utilized in societies.^30^

As mentioned in the introduction, a limitation of cost‐benefit analysis in radiation protection is that α‐values defined by authorities in other countries cannot directly be implemented in a given society.^8^, ^19^ One of the reasons is the difference in gross domestic product per capita between countries.^21^ In that context, an α‐value for the Swedish society in 2021 has been presented in the present study. Another limitation of cost‐benefit analysis in radiation protection (also mentioned in the introduction) is that cost‐benefit analysis is only useful when the relevant factors involved are possible to quantify in monetary terms, for example costs and collective dose.^9^, ^11^, ^12^, ^14^ In many cases within occupational radiological protection there are several factors to consider, and some of them may not be easy to quantify. For example, Huda et al.^6^ performed a cost‐benefit analysis of lead aprons in nuclear medicine in 1989. They reported that lead aprons were a good investment according to the recommended α‐value by ICRP at that time. They also addressed the risk that staff could experience a strained back due to wearing lead aprons for a long time, something that is difficult to quantify in monetary terms. If back pain is a relevant factor, when working with lead aprons for long time‐periods, it would influence the outcome in case 3.3 and case 3.6 in the present study. Therefore, one of the difficulties in cost‐benefit analysis is to decide which factors are relevant, and if these factors can be expressed in monetary terms. If there are factors that cannot be expressed in monetary terms, a decision aiding technic of a more qualitative nature is appropriate, for example multiattribute utility analysis.^11^, ^12^, ^14^ Another limitation is that cost‐benefit analysis is not an option when the occupational exposure is near or over the legislations of individual dose limits. For occupational radiological protection in Sweden, the α‐value interval recommended in the present study comes to best use when the individual doses are below 1 mSv.^12^, ^14^, ^50^

In ICRP Publication 101b,^13^ several topics related to the present work are discussed. For example, it is important to involve diverse stakeholders into the optimization process, which can be vital to identify all relevant factors. Furthermore, it is put forward that decision aiding technics such as cost‐benefit analysis is afflicted with both uncertainties and delineation of relevant factors and is in its core a judgmental process; transparency is therefore vital to the process to make informed decisions. Also, it is proposed that the optimization process should be seen as a frame of mind (the obligation of means) rather than focusing on specific outcomes of cost‐benefit analysis. Finally, it should be clarified that cost‐benefit analysis is a helpful tool for a decision‐maker, but it is not the answer to the correct decision.^11^, ^12^, ^13^, ^14^, ^18^ The responsibility of a decision is always left to the decision‐maker. For example (retrieved from case 3.7), how a controlled area is being used within a department of nuclear medicine can quickly change.In that context, it can be wise to install lead shielding of a drywall to meet future requirements.

It has been shown that the willingness to pay to prevent a radiation‐induced cancer death averted in Sweden varies with factor of 1000 between high and low.^19^ The goal with cost‐benefit analysis in radiation protection purposes is to narrow this interval. In many situations in cost‐benefit analysis, it is inevitable to set an economic value on human lives, which may be controversial.^8^ The answer to this criticism is that the resources available to society are finite, both in terms of natural resources and in manpower, and priorities must be made.^8^, ^12^ Here, alternative costs are important to consider, that is, what alternative activities could have been accomplished for the same monetary value. Again, the goal within health economics is to get the most social benefit out of investments in reducing risks in society. In this context, cost‐benefit analyses are already used in the Swedish nuclear industry^18^ and by several Swedish authorities and institutes, such as the Swedish Transport Administration^32^ and The Swedish Dental and Pharmaceutical Benefits Agency.^29^ A cost‐benefit analysis can show that a specific intervention in occupational radiological protection is reasonable in terms of cost effectiveness,^14^ which can be helpful for decision‐makers in the healthcare system. In other cases, interventions can be shown not to be reasonable in terms of cost effectiveness, and resources would then be better invested elsewhere within the healthcare system.

## 5 CONCLUSIONS

The aim of the present study was to demonstrate cases of cost‐benefit analysis within healthcare, of how economic factors can be considered in occupational radiological protection, in agreement with the ALARA principle and present Swedish legislations. An α‐value for the Swedish society in 2021 was presented as an interval between $45 and $450 per man mSv. The α‐value interval can be interpreted as following:

1. Less than $45 per man‐mSv is a good investment.
2. From $45 to $450 per man‐mSv, other factors than costs and collective dose are important to consider.
3. More than $450 per man‐mSv is too expensive.

The present case study shows a large variation in costs per collective dose from different types of occupational radiological protection, used at Skaraborg Hospital in Sweden.

## CONFLICT OF INTEREST

The authors declare that there is no conflict of interest that could be perceived as prejudicing the impartiality of the research reported.
